# Early-onset and rapid progression of arrhythmogenic cardiomyopathy in a pediatric patient with medium chain acyl-CoA dehydrogenase deficiency

**DOI:** 10.1016/j.hrcr.2026.04.007

**Published:** 2026-04-09

**Authors:** Tracie K. Lin, Nathaniel D. McConkey, Bryan P. Stefek, Jason R. Imundo

**Affiliations:** 1 University of Virginia Children’s Hospital, Charlottesville, Virginia; 2 Oregon Health and Science University, Portland, Oregon; 3 Penn State Health Children’s Hospital, Hershey, Pennsylvania

**Keywords:** Arrhythmogenic cardiomyopathy, Arrhythmogenic right ventricular cardiomyopathy, Pediatric, Ventricular tachycardia, Metabolic, Cardiac arrest, Medium chain acyl-CoA dehydrogenase deficiency

**Key Teaching Points**

• Atypical presentation, severity, or age-of-onset of arrhythmias for a patient with a known disease should prompt investigation into alternative etiologies.
• Unlike in long-chain fatty acid metabolism disorders, arrhythmias are typically rare in medium chain acyl-CoA dehydrogenase (MCAD) deficiency outside of metabolic crises.
• The diagnosis of arrhythmogenic right ventricular cardiomyopathy (ARVC) is partly informed by an understanding of the natural history and progression of the disease. Deviations from this typical progression, such as onset of arrhythmias much earlier in life or an unusually severe morphological phenotype, should prompt exploration of exacerbating comorbidities and inform decision making regarding risk stratification for sudden death.
• The pathophysiology of ARVC includes not only defects in the myocyte intercalated disk region, but also abnormalities in the canonical Wnt/β-catenin pathway which affects mitochondrial β fatty acid oxidation.
• Myocytes in patients with ARVC have been shown in experimental studies to demonstrate reduced MCAD expression and altered adipogenic signaling, suggesting one potential area of shared pathophysiology between ARVC and congenital MCAD deficiency.

## Introduction

Medium chain acyl-CoA dehydrogenase (MCAD) deficiency, the most common inherited disorder of fatty acid metabolism, is caused by variants in the *ACADM* gene located on chromosome 1p31. Pathologically low levels of this enzyme impair the use of fatty acids for ketogenesis and gluconeogenesis—particularly important for energy production during fasting or catabolic stress—and lead to accumulation of toxic metabolites that disrupt the urea cycle.^1^^,^^2^ In turn, patients with MCAD deficiency are at risk for metabolic crises during catabolic states, manifesting as severe hypoglycemia, hyperammonemia, emesis, lethargy, encephalopathy, seizures, respiratory arrest, or even sudden death.^1^

However, since the late 1990s most children with MCAD deficiency are diagnosed through newborn screening, allowing highly-effective preventative management. With appropriate dietary modifications, many patients with MCAD deficiency live asymptomatically.^1^ Additionally, unlike *long-chain* fatty acid oxidation disorders, MCAD deficiency is rarely associated with arrhythmias.^3^^,^^5^ Less than 30 years ago, it was thought that arrythmias did not occur with MCAD deficiency at all.^4^ Subsequent case reports have indeed documented arrhythmias, but these were associated with MCAD deficiency-related acute metabolic derangements. For example, before widespread newborn screening, previously-undiagnosed adults presented with multi-organ failure, supraventricular tachycardia, or ventricular fibrillation in the context of hypoglycemia and hyperammonemia.^6^ In recent years, however, arrhythmias are often only observed in neonates prior to diagnosis of MCAD deficiency, before preventative dietary modifications can be made.^3^^,^^7^^,^^8^ Corrected QT prolongation has been seen, but resolves once metabolic derangements are addressed,^7^ suggesting that the finding is because of electrolyte abnormalities rather than an inherent repolarization abnormality. It has also been hypothesized that accumulation of medium-chain acylcarnitines may be arrhythmogenic, which can occur acutely in patients with MCAD deficiency during attempted increases in fatty acid metabolism during catabolic stress.^9^

In contrast, arrhythmogenic cardiomyopathy—specifically arrhythmogenic right ventricular (RV) cardiomyopathy (ARVC)—is well-known to involve ventricular tachyarrhythmias. However, the fibrofatty replacement of myocardial tissue seen in ARVC, with an associated decrease in contractility and increase in ventricular arrhythmia risk, is typically slowly progressive, with malignant arrhythmias typically not seen until adulthood.^10^ Malignant arrhythmias are rare in prepubescent children.^11^ Research regarding ARVC pathophysiology has largely focused on desmosomes and other aspects of myocyte intercalated disk structure and function.^11^ However, other work has drawn attention to changes in transcription factor expression because of Wnt/β-catenin pathway abnormalities, with associated abnormalities intracellular lipid metabolism.^12^ Notably, this includes work that found decreased MCAD enzyme expression within the myocytes of patients with ARVC.^13^ However, to our knowledge, as of the time of publication of this case report, there has not yet been documentation of a clinical case demonstrating the effects of co-occurrence of ARVC with congenital MCAD deficiency.

Here, we present a case of a young patient with congenital MCAD deficiency with the atypical finding of new-onset malignant ventricular dysrhythmias in his early teenage years, who on further workup was also found to have findings consistent with highly-advanced ARVC.

## Case report

**Figure 1:**
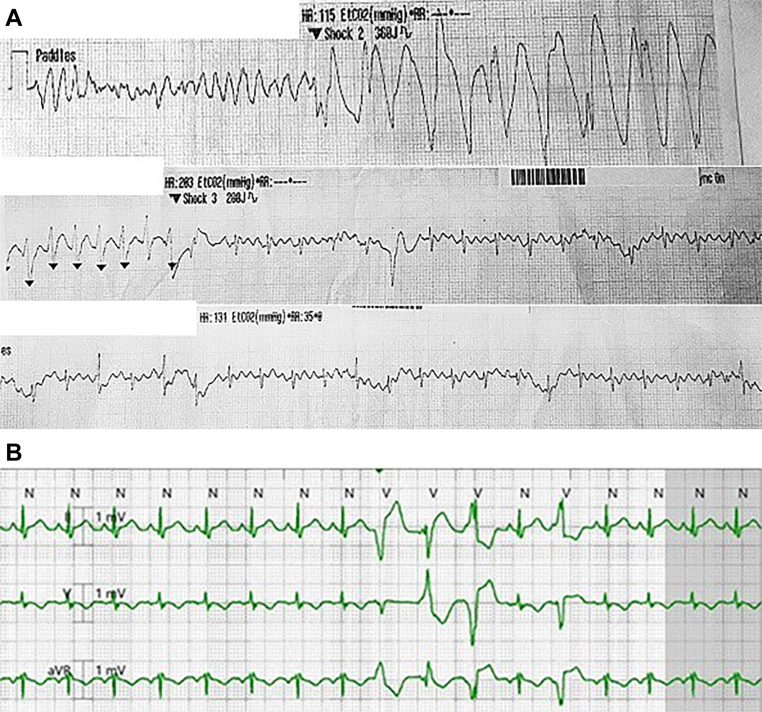
**A:** Rhythm strips provided by emergency medical services (EMS). **B:** During admission, his baseline rhythm was consistently sinus rhythm, with intermittent polymorphic ventricular ectopy with up to couplets and triplets including ventricular/fusion beats.

The patient was a 13-year-old male with MCAD deficiency diagnosed by routine newborn screening. His MCAD deficiency was well-managed at home by regular meals and avoiding any prolonged fasting, glucose checks with any illness and fluid hydration with higher sugar concentrations during gastro-intestinal illnesses. With this home management strategy there was no prior history of metabolic crises. He had no known preexisting cardiac problems and reported being very active in recreational sports and regular exercise. Beyond the routine cardiac examination by his primary care physician, more extensive cardiac evaluation had not been performed as such evaluation outside of the neonatal period in asymptomatic patients is not currently part of the routine management recommendations for MCAD deficiency. On the day of presentation, he was in his usual state of health when he acutely experienced palpitations and chest pain while watching television at home. He fell to the ground, was noted to be pulseless, and received immediate bystander cardiopulmonary resuscitation. Emergency medical services was activated and arrived at the home in 7 minutes. He was found to be in ventricular fibrillation and received defibrillation, additional cardiopulmonary resuscitation, then defibrillation again for ongoing ventricular fibrillation (Figure 1A). Return of spontaneous circulation was obtained, followed by ventricular tachycardia for which he underwent synchronized cardioversion, finally converting to an apparent sinus rhythm. He was intubated in the field. En route to the local emergency department, he had an episode of seizure-like activity for which lorazepam was given.

He was subsequently transferred to our quaternary care pediatric hospital for further management. Urinary toxicology screen, electrolyte panel, liver function studies, complete blood count, and thyroid studies were all unremarkable. N-terminal pro-B-type natriuretic peptide and troponin T levels were observed to be 320 pg/mL (reference range <125 pg/mL) and 0.174 ng/mL (reference range <0.010), respectively. Electroencephalogram did not show any evidence of seizures, and his brain magnetic resonance imaging (MRI) was unremarkable.

**Figure 2:**
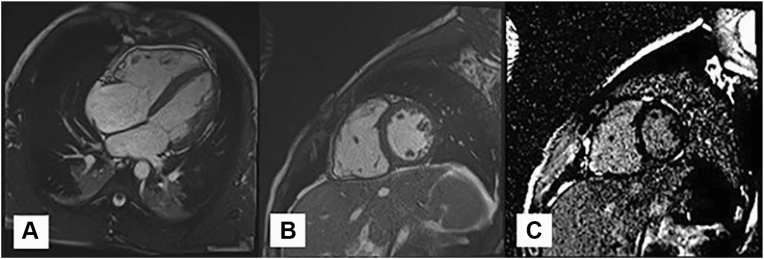
Cardiac MRI on hospital day 4 demonstrating (**A)** enlarged RV with systolic antero-inferior regional wall dyskinesia in 4-chamber gradient echo sequence; (**B)** diastolic wall motion abnormality in short axis gradient echo sequence; and (**C)** late gadolinium enhancement in area of wall motion abnormality on phase-sensitive inversion recovery (PSIR) sequence. MRI = magnetic resonance imaging; RV = right ventricular.

During his hospitalization, he had intermittent polymorphic ventricular ectopy (Figure 1B) and was started on sotalol 80 mg twice daily based on his weight of 60 kg. His baseline 12-lead electrocardiogram in normal sinus rhythm demonstrated left axis deviation, non-specific inferior T wave changes. On chart review, it was noted that his carnitine profile performed 3 weeks prior showed a low free carnitine level of 17 umol/L (reference range 22–63 μmol/L) and elevated carnitine esters of 41 μmol/L (reference range 3–38 μmol/L), consistent with MCAD deficiency. Cardiac MRI demonstrated RV enlargement (indexed RV end diastolic volume 151 mL/m^2^), global systolic dysfunction with a severely reduced RV ejection fraction of 25%, with paradoxical motion of the mid-anterior basilar RV free wall and dyskinesia in the anterior and mid right ventricular outflow tract. There was focal late gadolinium enhancement in the region of dyskinesia, consistent with fibrosis (Figure 2). These findings meet a major structural criterion under the 2010 Task Force Criteria for arrhythmogenic right ventricular cardiomyopathy, based on the presence of regional RV wall motion abnormalities with severe RV systolic dysfunction. At follow-up after age 14 years, the patient developed T-wave inversions in leads V_1_–V_4_ with a terminal activation duration of the QRS >55 ms, constituting a major electrocardiographic criterion, thereby fulfilling 2 major criteria consistent with a definite ARVC diagnosis.^14^

Given the severity of his presentation, it was determined that an intracardiac defibrillator (ICD) for secondary prevention was indicated during the index hospitalization. He underwent implantation of a single chamber transvenous ICD on day 8 post-arrest. During RV lead placement, most of the endocardium yielded only low-voltage electrograms. Several attempts at repositioning were required before a region of the RV septum with an amplitude over 4 mV could be identified. An RV tip-to-coil vector was chosen to maximize the region of viable tissue included in the sensed field, and defibrillation threshold testing was performed to ensure adequate arrhythmia sensing and delivery of therapy. The final lead position eventually yielded a sensing amplitude over 6 mV at 3-month follow-up. For a provoked lower extremity deep vein thrombosis in the setting of a central line while in the pediatric intensive care unit, he was also managed with apixaban and fortunately did not experience any bleeding complications from his ICD placement. He was also started on carnitine supplementation.

At outpatient follow-up, a comprehensive 168-gene arrhythmia and cardiomyopathy genetic testing panel revealed heterozygosity for a variant in plakophilin-2 (PKP2), c.1517T>C (p.Leu506Pro). At the time, our patient’s particular missense variant was considered of uncertain significance, but it has also been implicated in a prior case report of a family with ARVC,^15^ and our patient’s personal clinical presentation and MRI findings were highly suspicious for the variant contributing to his clinical presentation. According to American College of Medical Genetics and Genomics (ACMG)/Association for Molecular Pathology variant interpretation guidelines, the variant remains classified as a variant of uncertain significance (VUS). The variant is extremely rare in population databases (gnomAD allele frequency approximately 0.003%). Computational prediction algorithms suggest a deleterious effect on protein structure and function (PP3), and the variant has been previously reported in individuals with arrhythmogenic right ventricular cardiomyopathy. However, additional supporting evidence such as segregation data or functional validation is currently lacking, preventing definitive reclassification. Our team advised the patient’s first-degree relatives to undergo genetic screening as well, but the family opted to not follow this advice.

## Discussion

This 13-year-old patient’s findings were unusual in their severity for either MCAD deficiency or ARVC in isolation. Malignant ventricular arrhythmias and the degree of fibro-fatty myocardial replacement seen in our patient is not typically seen in ARVC until adulthood.^11^ Likewise, in the absence of an acute metabolic crisis, arrhythmias are usually uncommon for patients with good dietary control of their MCAD deficiency.^3^^,^^8^ We hypothesize that our patient’s atypical phenotype may have been affected by shared metabolic and cellular pathophysiology between MCAD deficiency and his newly-diagnosed, concurrent ARVC.

**Figure 3:**
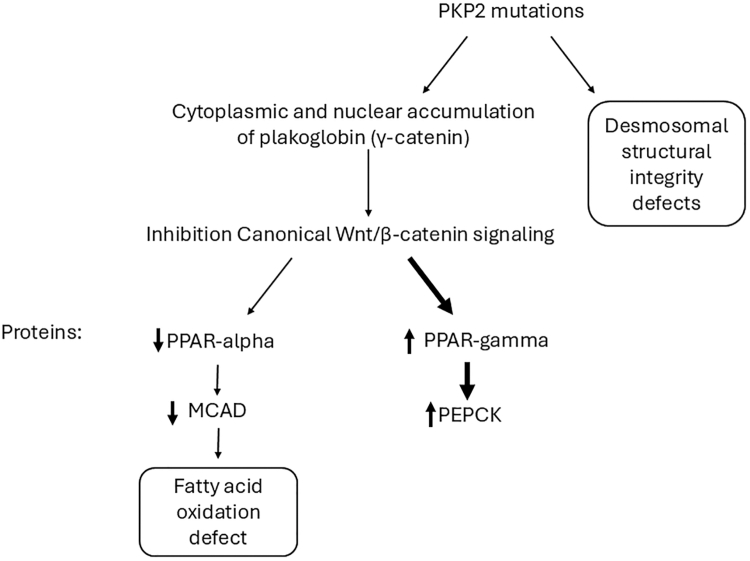
PKP2 is a structural desmosomal protein that has also been implicated in signaling pathways involving plakoglobin and the Wnt/β-catenin pathway. Dysregulation of these pathways has been associated with altered adipogenesis and reduced expression of metabolic enzymes such as MCAD in cardiomyocytes. MCAD = medium chain acyl-CoA dehydrogenase; PKP2 = plakophilin-2.

Arrhythmogenic cardiomyopathy is primarily a genetic disease associated with variants affecting desmosomal proteins. These abnormalities lead to weakening of intercellular adhesion between cardiomyocytes, electromechanical uncoupling, and progressive myocyte loss with fibrofatty myocardial replacement, ultimately predisposing patients to ventricular arrhythmias, sudden cardiac death, and ventricular dysfunction. The pathophysiology of ARVC is complex and has been the subject of extensive investigation. More than 11 gene loci have been found to be associated with the disease to date, inherited in both autosomal dominant and recessive forms. The great deal of work into ARVC pathophysiology has focused on defects in myocyte cell-to-cell interaction.^11^ However, another important line of inquiry is regarding the canonical Wnt/β-catenin pathway involved in adipogenesis and peroxisome proliferator-activated receptor (PPAR) signaling, which has been implicated in multiple cardiovascular diseases including ARVC.^11^, ^12^, ^13^^,^^16^^,^^17^ (Figure 3) PPAR-gamma and PPAR-alpha are transcription factors involved in cardiac ventricular myosin function and adipogenesis. Importantly, one downstream target of these transcription factors is the gene for the MCAD enzyme, and myocytes of patients with ARVC have been found to have decreased activation of the PPAR-alpha pathway with resultant lower production of MCAD messenger RNA and proteins compared with controls. This has been found to correspond to impaired myosin sliding, consistent with the decreased contractile function seen clinically.^13^ One could hypothesize that the effects of PKP2 abnormalities on desmosomal structural integrity, combined with its effects on regulation of MCAD enzyme expression, could further compound diminished MCAD enzyme availability because of an inherited ACADM-related MCAD deficiency, as in our patient. It is unknown whether our patient’s underlying MCAD deficiency accelerated his ARVC-associated fibro-fatty myocardial replacement—by way of concurrent fatty acid metabolic dysregulation and myocyte structural compromise—with resultant accelerated ventricular damage and arrhythmogenicity.

Turning one’s attention to altered fatty acid oxidation within cardiac myocytes as part of the underlying pathophysiology of both MCAD deficiency and ARVC opens the potential for overlapping treatment options for patients in both groups.^12^ From a clinical management standpoint, avoidance of catabolism via dietary modification is a cornerstone of preventative care for patients with fatty acid oxidation disorders, with the goal of averting metabolic crises and the accumulation of toxic fatty acid metabolites.^1^ Patients with MCAD deficiency are also advised to avoid aspirin, which can exacerbate metabolic derangements by increasing mitochondrial fatty acid oxidation.^6^ Carnitine supplementation can also be considered in the setting of carnitine deficiency secondary to MCAD deficiency.^1^ Whether dietary strategies, carnitine supplementation, and avoidance of aspirin can mitigate the progression of cardiomyopathy-associated myocyte steatosis, progressive myocardial dysfunction, and/or arrhythmogenicity in ARVC patients may be worth investigating. On the research forefront, attention to the Wnt/β-catenin pathway’s role in cardiovascular disease has led to exciting work in multiple preclinical models.^17^ Augmenting activation of the Wnt/β-catenin pathway using a novel compound, SB216763, a glycogen synthase kinase-3β inhibitor, has been demonstrated to prevent or actually reverse the cardiac structural and functional abnormalities seen in a murine model of ARVC.^16^

As for potential improvements in MCAD deficiency management, phenotypes of MCAD deficiency are highly variable, making organ-specific screening recommendations potentially challenging because of significant individual differences over the course of patients’ lifetimes. Currently, aside from cardiologist evaluation being a consideration for the newly-diagnosed neonate,^6^ repeated follow up cardiac screening or imaging later in life is not yet considered part of routine management for patients found to be genotype positive for MCAD deficiency. However, a case report that included post-mortem histopathology of a patient with MCAD deficiency demonstrated microvesicular steatosis and vacuolation of the cardiac myocyte cytoplasm,^3^ similar to that seen in patients diagnosed with cardiomyopathy.^12^ It is unknown whether in other patients with MCAD deficiency, routine cardiac screening performed outside of the newborn period would also lead to the finding of late gadolinium enhancement abnormalities on cardiac MRI akin to that seen in primary cardiomyopathies.

Finally, our patient had a missense variant in the PKP2 gene that was considered a VUS, c.1517T>C (p.Leu506Pro).^15^ We performed formal ACMG/Association for Molecular Pathology variant classification. The PKP2 c.1517T>C (p.Leu506Pro) variant is present at extremely low frequency in population databases (gnomAD allele frequency ∼10^-6^ to 10^-5^), supporting PM2. Multiple in silico prediction tools and high evolutionary conservation (phyloP100 7.8) support a deleterious effect (PP3). The patient meets definite 2010 Task Force Criteria for ARVC, supporting phenotype specificity (PP4). In the absence of segregation or functional data, the overall evidence remains insufficient to reclassify the variant beyond VUS under ACMG guidelines. Because the family opted not to pursue genetic testing of relatives, we were unfortunately unable to perform co-segregation analysis.

In summary, although it is unknown if overlapping metabolic pathophysiology between MCAD deficiency and ARVC contributed to our patient’s unique presentation, we hope that our clinical case report helps encourage additional work regarding shared aspects of pathophysiology behind both cardiomyopathies and MCAD deficiency—namely interactions between desmosomal structural regulation and the Wnt/β-catenin pathway’s role in cardiomyocyte lipogenesis.

## Conclusion

There is significant phenotypic variation in both MCAD deficiency and ARVC, and much to elucidate about their underlying mechanisms for arrhythmogenesis. Our case draws attention to multiple potential areas for further inquiry, namely intracellular lipid metabolism abnormalities in ARVC, and subclinical myocardial tissue abnormalities in patients with MCAD deficiency—and what those shared pathophysiological mechanisms may imply about options for preventative management and therapies for both diseases.

## Disclosures

The authors have no conflicts of interest to disclose.
